# Reward Draws the Eye, Uncertainty Holds the Eye: Associative Learning Modulates Distractor Interference in Visual Search

**DOI:** 10.3389/fnbeh.2017.00128

**Published:** 2017-07-11

**Authors:** Stephan Koenig, Hanna Kadel, Metin Uengoer, Anna Schubö, Harald Lachnit

**Affiliations:** Department of Psychology, Philipps-Universität Marburg Marburg, Germany

**Keywords:** associative learning, attention, oculomotor capture, reward, uncertainty

## Abstract

Stimuli in our sensory environment differ with respect to their physical salience but moreover may acquire motivational salience by association with reward. If we repeatedly observed that reward is available in the context of a particular cue but absent in the context of another cue the former typically attracts more attention than the latter. However, we also may encounter cues uncorrelated with reward. A cue with 50% reward contingency may induce an average reward expectancy but at the same time induces high reward uncertainty. In the current experiment we examined how both values, reward expectancy and uncertainty, affected overt attention. Two different colors were established as predictive cues for low reward and high reward respectively. A third color was followed by high reward on 50% of the trials and thus induced uncertainty. Colors then were introduced as distractors during search for a shape target, and we examined the relative potential of the color distractors to capture and hold the first fixation. We observed that capture frequency corresponded to reward expectancy while capture duration corresponded to uncertainty. The results may suggest that within trial reward expectancy is represented at an earlier time window than uncertainty.

## Reward Expectancy, Reward Uncertainty, and Attention Bias

Humans inhabit rich sensory environments in which the abundance of sensory information is difficult to apprehend and act upon at once. In such environments, selective attention provides the vital ability to select some stimuli for enhanced processing at the cost of neglecting others. As criticized by Awh et al. (2012), attention research has too long focused on the conceptual dichotomy of bottom-up (exogenous) vs. top-top (endogenous) processing where the potential of external stimuli to attract attention is determined by their physical salience and task relevance respectively (Wolfe et al., 1989; Desimone and Duncan, 1995; Itti and Koch, 2001; Corbetta and Shulman, 2002). In support of this critique, numerous recent studies have provided evidence that attention can be captured automatically by non-salient and currently task-irrelevant stimuli if these stimuli have a previous learning history of association with reward (Anderson et al., 2011a,b; Anderson and Yantis, 2012; Theeuwes and Belopolsky, 2012; for review, see Anderson, 2015). In these experiments, the capture of attention was measured as the potential of a reward-associated but task-irrelevant color distractor to slow visual search for a shape target. For example, in the first stage of the experiment of Anderson et al. (2011b), participants were instructed to search for a circular target rendered in one of two target-defining colors (e.g., red or blue) and to ignore all distractors rendered in different colors. The task of the participants was to report the orientation of a line embedded within the target and they received monetary reward for a correct response. The amount of reward, however, was dependent on the target color. For one target color, participants received a high reward in 80% of the trials and a small reward in 20% of the trials while the reverse reward contingencies (20% high, 80% small) were true for the other target color. In a subsequent test stage, participants were instructed to search for a shape singleton (diamond) embedded amongst circular distractors of varying colors. Despite the fact that colors now were irrelevant and participants could ignore them, presence of a high reward distractor significantly slowed reaction times to the shape target. Furthermore, Anderson et al. (2011a) and Anderson and Yantis (2013) demonstrated that value driven capture was more pronounced for colors previously associated with a large reward than for colors associated with a smaller reward.

While the above studies used the latency of the manual response to the shape target to infer the effect of learned value on covert attention, other studies employed eye tracking to examine overt attention. For example, Anderson and Yantis (2012) demonstrated reward-driven capture in an unconstrained viewing version of the search task in which participants were allowed to move their eyes. Under this condition, a previously rewarded color distractor was more likely to attract ocular fixations than an equally salient color distractor not associated with reward. For onset distractors, Theeuwes and Belopolsky (2012) demonstrated that the rate of oculomotor capture again was higher for high reward distractors than low reward distractors.

**Figure 1:**
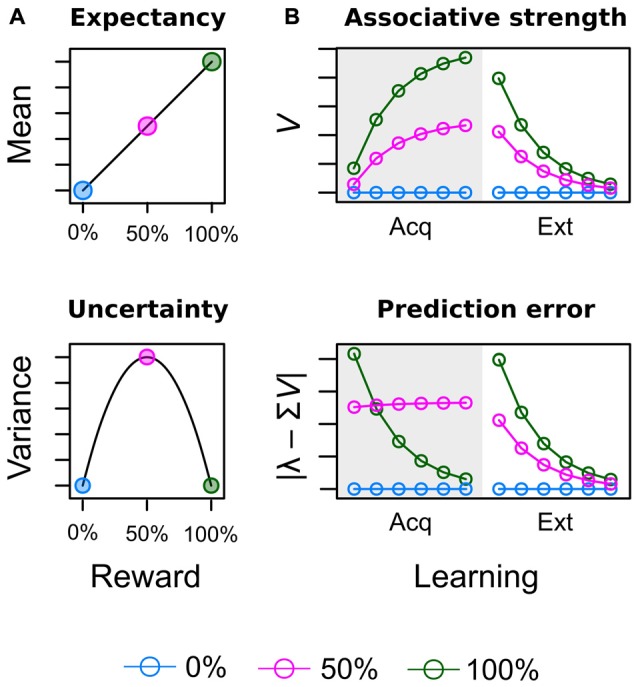
Illustration of acquired values for a conditioning experiment in which three different cues are followed by reward on 0% [blue], 50% [red] and 100% [green] of the trials. **(A)** In neuro-economic theory (Schultz et al., 2008), reward expectancy and uncertainty (risk) are computed as the mean and variance of the reward’s probability distribution. **(B)** In associative learning theory (Rescorla and Wagner, 1972) expectancy corresponds to the strength of the learned cue-reward association *V*. Uncertainty corresponds to the absolute prediction error caused by the cue | λ – Σ*V*|, where λ represents the actual outcome and Σ*V* represents the predicted outcome as further detailed in the Supplementary Material Appendix.

Theoretical considerations in the field of study summarized above do acknowledge the fact that attentional bias is shaped by learning history but provide no formal account of how reward value is computed in the first place. Figure 1 illustrates such computations from the perspectives of neuro-economic theory (Figure 1A; Schultz et al., 2008) and associative learning theory (Figure 1B; Rescorla and Wagner, 1972) for an experiment in which three different cues were followed by reward on 0%, 50% and 100% of the trials respectively (see Supplementary Material Appendix A for simulation details). Both perspectives acknowledge the importance of two key variables to encode the learned values of the cues. In economic theories, these are *reward expectancy* and *reward uncertainty* and are computed as the mean and variance of the reward’s probability distribution respectively (Schultz et al., 2008). Reward expectancy linearly increases with the average amount of associated reward (0% < 50% < 100%) as shown in the top panel of Figure 1A. On the other hand, reward uncertainty for the 50% reinforced cue will exceed uncertainty elicited by the continuously (non-) reinforced 0% and 100% cues as shown in the bottom panel. Figure 1B depicts how equivalent values are computed in the associative learning theory of Rescorla and Wagner (1972) as the “most widely accepted description of associative changes during classical conditioning” (Gluck and Bower, 1988, p. 228). The theory proposes that after each learning episode reward associations (top panel) are updated based on the observed prediction error (bottom panel) i.e., the difference between the predicted and actual reward. When reward contingencies of 0%, 50%, and 100% are in effect in the acquisition stage (gray shaded area), associative strength and prediction error are driven towards economic expectancy and uncertainty respectively. At the outset of learning, the highest error is registered for the 100% cue because it had no prior association with reward but now is continuously rewarded. This registered error of “more reward than expected” increases the reward association which in turn decreases the error of future learning episodes. Learning approaches a stable asymptote when reward is perfectly anticipated and the error finally becomes zero. In contrast, for an uncertain cue partially reinforced at a rate of 50% the prediction error is retained at a high level because even at the end of training the prediction is always off by half the outcome’s value. For example, for random payment of either 0 or 10 Cent, the asymptotic prediction of 5 Cent is always off by ±5 Cent.

Most previous studies on value-based capture have focused on associated reward (high vs. low reward) but neglected the possibility that reward uncertainty (prediction error) may also affect attentional processing. From competing perspectives, this hypothesis has been formalized in the associative learning theories of Mackintosh (1975); Kruschke (2001); Pearce and Hall (1980) and Pearce et al. (1982). Mackintosh proposed more *attention to predictive cues* (0% or 100% reward probability) than uncertain cues (50% reward probability) whereas Pearce and Hall proposed more *attention to uncertain cues* (50% reward probability) than predictive cues (0% or 100% reward probability). We conducted the current experiment to provide further support for the idea of Pearce and Hall that partial reinforcement should increase attention to uncertain cues of reward as suggested by previous animal learning experiments (Kaye and Pearce, 1984; Swan and Pearce, 1988; Haselgrove et al., 2010), and human eye tracking studies (Hogarth et al., 2008; Beesley et al., 2015). In extension to this existing evidence, our current experiment adopted the value-based capture paradigm to examine the question whether reward expectancy and uncertainty acquired in one task (learning task) would transfer to a second task (search task) in which reward-associated stimuli were introduced as irrelevant distractors. In the *learning task* two different colors were consistently paired with high reward (H; 10 Cent) and low reward (L; 1 Cent) respectively while a third color was partially reinforced (P) and followed by high reward in 50% of the trials (and no reward in the remaining trials) to induce uncertainty. In the *search task*, colors were introduced as task-irrelevant distractors while participants searched for a shape target, and we measured the potential of the trained color distractors to capture and hold overt attention. Our hypotheses were derived from the theoretical values shown in Figure 1. If attention is driven be reward expectancy the interference by color distractors in the search task should monotonically increase with the average amount of associated reward, L < P < H. In contrast, if attention is driven by uncertainty (prediction error), the color with 50% reward probability in the learning task should induce more interference than either the low or high reward cues, L < P > H.

Our focus on reward expectancy *and* uncertainty required some adjustments to the original design of Anderson et al. (2011a,b) and Anderson and Yantis (2012). These previous experiments presented learning trials and search trials in two consecutive blocks, where the blocked presentation of unrewarded search trials after the initial learning stage basically constituted a phase of continuous extinction of reward associations. Accordingly, some experiments yielded response differences between low and high valued distractors during the first few hundred search trials only while with further extinction these differences vanished (e.g., Anderson et al., 2011a, Experiment 1; Anderson et al., 2016). The right-hand side of the upper panel in Figure 1B depicts the effects of such extinction (Ext) of reward associations in the Rescorla-Wagner model. While the exact speed of extinction is dependent on the learning rate (a free parameter), persistent attentional effects of reward during extinction can be attributed to the fact that the *rank order of reward associations* is preserved during the entire extinction stage before all cues eventually converge towards zero. Former cues for high reward thus may attract more attention because their association with reward still exceeds the reward association of low reward cues at any pre-asymptotic point during extinction. Inspection of the bottom panel in Figure 1B makes clear that the same preservation of rank order does not exist for the prediction error. Towards the end of the acquisition stage, cues with a reward probability of 50% persistently cause a higher prediction error than cues with reward probabilities of either 0% or 100%. During extinction, however, the new outcome of each cue is the omission of reward and the cue that was always followed by reward previously now causes a higher prediction error (difference between 100% expectancy and no actual reward) than the previously partially reinforced, uncertain cue (difference between 50% expectancy and no actual reward). In turn, we cannot examine if an attentional bias is caused by reward expectancy or uncertainty when both values form the same rank order during extinction. To remedy this problem our experiment featured several adjustments to the attentional capture paradigm to prevent extinction and instead maintain values of expectancy and uncertainty from the acquisition stage.

### Intermixed Learning Trials and Search Trials

In contrast to previous studies, search trials were not presented in a separate block after the acquisition of reward associations but rather were randomly interspersed with learning trials to avoid a continuous block of extinction trials. During the intermixed presentation of trial types, participants were instructed ahead of each trial whether the next trial would be a learning trial or a search trial.

### Different Colors in Learning Trials and Search Trials

In contrast to previous studies, we used different colors during learning and search. Participants thus never experienced that the same color rewarded in learning trials was unrewarded in search trials. Rather, the transfer from learning to search occurred because of the similarity of colors. For example, if two different red colors were associated with a high reward during learning, the red color used in search trials was not identical to either but was equally similar to both (in CIE L*a*b color space). Such transfer has not yet been demonstrated for value-based attentional capture in particular but is strongly suggested by the overall idea of response generalization in associative learning (Pearce, 1987; Shepard, 1987)^1^.

### Different Responses during Learning and Search

In previous versions of the task participants performed the same manual response during learning trials and search trials. They experienced that reward was paid after a correct response in the context of a specific color cue during learning but that reward was omitted for the same response in the context of the same color during search. In contrast, in our experiment participants responded with their left hand to color cues during learning trials while a right-hand response to the shape target was required during search. Participants thus experienced search trials not only as trials where reward was not available but rather as trials where a potentially rewarded left hand-response was inadequate in principle.

### Uncertainty about Reward and Response

Our experiment required the learning of color-response associations in order to gain reward. For example, in the learning task participants had to associate two red colors with two different left-hand buttons and for both colors a correct response earned high reward. While this color-response mapping was consistent in the predictable high and low reward conditions, it was random in the partial reward condition. Uncertainty thus was induced: (a) with reference to which button to choose; and in turn (b) with reference to how much reward to expect. Importantly, this response uncertainty was protected from extinction during search trials because as outlined above: (a) participants did not perform left-hand responses in the search task; and (b) the search task did not include the exact colors trained with specific responses in the learning task.

## Materials and Methods

The study was approved by the local ethics committee of the Department of Psychology at the University of Marburg (AZ: 2013-28k). All subjects gave written informed consent in accordance with the Declaration of Helsinki.

### Participants

Thirty-three students of the University of Marburg participated in the experiment and received either course credit or payment. All participants had normal or corrected-to-normal vision. Since correct responding in the learning task was essential for establishing the intended reward contingencies (low, partial, high) we conducted a manipulation check by analyzing the performance of individual subjects in the learning task. A density plot of the response accuracies revealed a bimodal distribution with two distinct subgroups. The accuracy in a subgroup of nine *poor learners* was at chance level or below (37%–53% correct, *M* = 46.3, *SE* = 1.67). For these nine subjects the trained colors cannot be expected to be associated with different values of reward expectancy and uncertainty and they were excluded from further analysis. In the remaining group of 24 *good learners* the percentage of correct responses was in the range of 64%–98% (*M* = 81.3, *SE* = 2.182). Eighteen of the good learners were female and six were male. Their age ranged from 20 to 27, *M* = 23.04, *SD* = 1.98.

### Stimuli and Apparatus

Testing took place in a sound-attenuated, dimmed room. Monocular eye movements were recorded using an infrared video-based eye tracker (Eyelink 2000, SR-Research, Mississauga, ON, Canada) that sampled gaze position at a frequency of 1000 Hz. Sampling of the left vs. right eye was counterbalanced across participants. The eye tracker restrained the participants head via chin and forehead rests and was table-mounted in front of a 22″-CRT monitor (Iiyama, Vision Master Pro514) that was color-calibrated using the eye-one display2 colorimeter (GretagMacbeth)^2^. Eye-to-screen-distance was 78 cm. The eye tracker was calibrated with a 9-point grid of calibration targets. For each participant, the calibration procedure was repeated until subsequent validation confirmed a maximal calibration error <0.5°. Stimulus delivery was controlled by Presentation® software (Version 16.1^3^).

**Figure 2:**
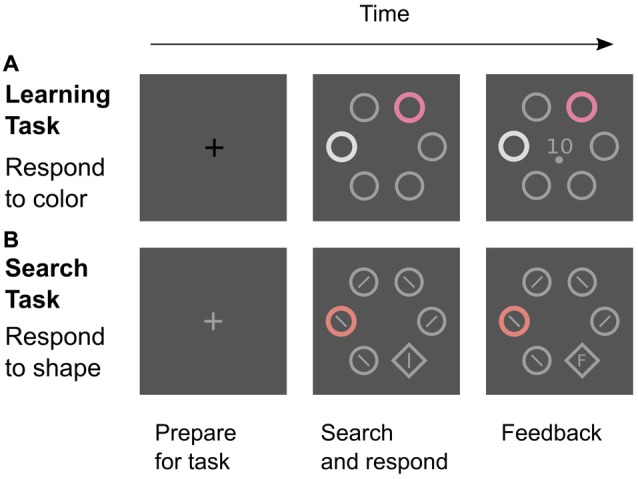
Two tasks in the experiment. **(A)** Learning trials were signaled by a black fixation cross. Participants attended to the color cue and made a left-hand choice response (upper or lower button) that was rewarded if correct. The feedback screen showed the amount of reward (0, 1, or 10 Cent) and the required response (upper or lower dot). **(B)** Search trials were signaled by a gray fixation cross. Participants were instructed to find and look at the shape target as quickly as possible and to ignore any color distractor. After a right-hand response to the line orientation within the target (left or right button), the reaction time or an “F” (for “False”) was displayed inside the diamond as feedback. All screens were shown for 2 s.

The experiment featured two different types of trials: *learning trials* to establish color-reward associations and *search trials* to test for attentional effects of these associations (Figure 2). In both tasks, the stimuli were positioned in a circular search array consisting of six stimuli that were placed at a distance of 100 mm (7.34 degrees of visual angle; dva) from the center of the computer screen. In learning trials, the search array consisted of four gray distractor annuli, one white distractor annulus, and one colored annulus that was the relevant cue for a rewarded manual response. In search trials, the relevant target stimulus was a gray shape singleton (diamond) that was presented amongst five distractor annuli which all were gray except for one colored distractor annulus that was similar to a reward-associated color from the learning task. This color distractor was presented in 75% of all search trials. The annuli (the diamond) measured 31 mm (34 mm) in diameter. Gray distractor annuli were drawn with a line width of 2 mm while all other stimuli (diamond, colored, and white annulus) were drawn with a line width of 4 mm.

**Figure 3:**
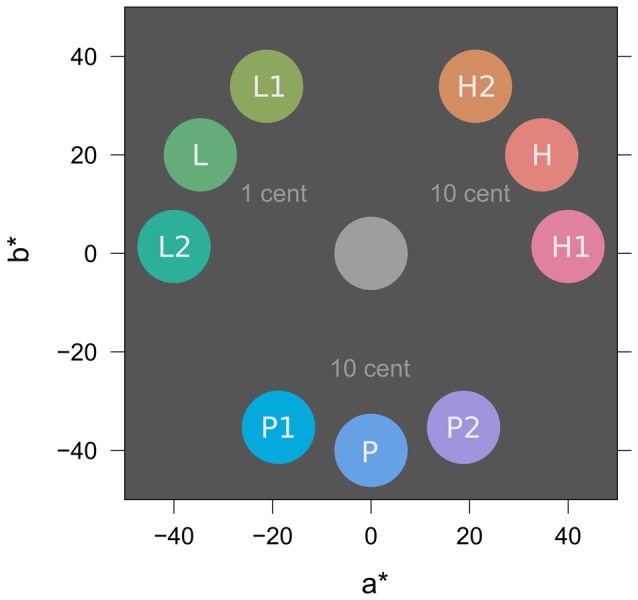
Stimulus colors in CIE L*a*b* color space (McLaren, 1976; International Commission on Illumination, 2004). L* specifies lightness (set to L* = 65 for all colors), a* and b* represent opponent colors green-red and blue-yellow respectively. In-text references to colors use the polar version of this color space with parameters lightness (L*, 0–100), chroma (C*, 0–100), and hue (h°, 0–360). For illustration, the figure shows a possible assignment of specific colors to experimental conditions of low (L), partial (P), and high (H) reward; this assignment was counterbalanced across participants. Rewarded colors in the learning task were L1, L2, P1, P2, H1, and H2. Distractor colors in the search task were L, P, and H and acquired value because of their similarity to colors in the learning task. For further explanation see text.

Figure 3 depicts colors from the experiment in CIE L*a*b* color space (McLaren, 1976; International Commission on Illumination, 2004). All colors were matched for lightness (L* = 65) and chroma (C* = 40). In the learning task, two similar colors H1 and H2 (e.g., both red; h° = 2, 58) were associated with a high probability of high reward (10 Cent). Two similar colors L1 and L2 (e.g., both green; h° = 122, 178) were associated with a high probability of low reward (1 Cent) and two similar colors P1 and P2 (e.g., both blue; h° = 242, 298) were associated with partial reinforcement (random payment of either 0 or 10 Cent) to induce reward uncertainty. In the search task, distractor colors H (red; h° = 30), L (green; h° = 150), and P (blue; h° = 270) were located at the perceptual center between the similar two hues from the learning task to promote equal generalization from L1 and L2 to L, from P1 and P2 to P, and from H1 and H2 to H, respectively.

### Procedure

As described in the previous section, colors in the learning task were associated with low reward (L), partial reinforcement (P), and high reward (H), and we tested whether acquired values of reward expectancy and uncertainty transferred to the search task to influence attentional capture by similar colors. As established in the introduction, learning trials and search trials were not presented in two consecutive blocks but rather were presented randomly intermixed to avoid a continuous phase of extinction caused by unrewarded search trials. During the experiment, participants were informed about the type of the next trial by the color of the initial 2-s fixation cross as depicted in Figure 2. A black cross signaled an impeding learning trial while a light-gray cross signaled an impeding search trial. The following sections give further detail on both tasks.

#### Learning Trials

In learning trials, the 2-s fixation cross was followed by a 2-s circular search display containing the color cue. The six different colors presented across learning trials differed with respect to: (a) which response button to press to gain reward; (b) the uncertainty in choosing the correct button; and (c) the amount of expected reward. For example, in the high reward condition one red color H1 consistently indicated that Button 1 was correct (upper button with left hand index finger), whereas after the other red color H2 Button 2 was correct (lower button with left hand thumb). If participants responded correctly in these trials they were paid a reward of 10 Cent at the end of the trial. Reward was omitted after an incorrect response. In the low reward condition, two different colors L1 and L2 in the same way were reliable cues for which button to choose (L1 → Button 1, L2 → Button 2), but the maximum reward that could be earned in a single trial was only 1 Cent. Trials H1/H2 and L1/L2 thus differed in the amount of associated reward but all were equally good cues for: (a) which button to press; and (b) how much reward to expect. In contrast to these predictable conditions, we used partial reinforcement to implement conditions of high uncertainty for cues P1 and P2. These two colors gave no information about which button to choose, since after both colors each response was correct in 50% of the trials. If, however, participants were correct by chance they were paid 10 Cent as in the high reward condition. With reference to our simulation in Figure 1, cues L, P, and H thus had contingencies of 0%, 50%, and 100% with the high reward outcome of 10 Cent in an ideal learner that always chose the correct button in the predictable conditions.

The display containing the color cue was shown for 2 s and a response was valid during the whole interval. After 2 s a feedback screen was shown that displayed the amount of earned reward (0, 1, or 10 Cent) as well as which button (lower, upper) was correct (indicated by a gray dot above or below the reward value). The feedback screen was shown for 2 s, and the next trial started after a random pause of 1–3 s showing an empty screen.

Written instruction about the learning task were presented prior to the experiment. Participants were informed: (1) that all learning trials began with a dark fixation cross; (2) that the color cue in the display could inform them about which button (upper or lower) to press with their left hand; (3) that they had to learn which color instructed which button during the experiment; (4) that the feedback screen would show the amount of monetary reward that was earned in that trial; and (5) that they would receive the total amount of earned reward at the end of the experiment. Twelve practice trials were administered prior to the experiment (showing a black cue instead of a color cue) to make sure that participants understood the instructions for the learning task.

#### Search Trials

After the first two blocks of training, search trials were presented interspersed with learning trials in Block 3 to Block 6. All search trials began with a light gray fixation cross that instructed participants that the subsequent search display would require selection of the shape singleton (diamond) while any colored annulus could be ignored. When the search display was presented, participants were required to find and to fixate on the shape target as quickly as possible and to indicate the line orientation in the target by pressing a right vs. left mouse button with their right hand. The search display could contain only gray distractors (baseline condition) or one of three color distractors associated with high, partial, or low reward. As outlined above, distractor colors in the search task were not identical to trained colors but rather marked the perceptual center of the two colors trained in the same experimental condition (see Figure 3). Note that when the color distractors from the high and low reward conditions (H and L) were presented in the search task, these colors were not expected to bear a dominant association with either Button 1 or 2 from the learning task although colors H1, H2, L1, and L2 were trained to acquire these associations. If colors in the search task elicited some form of automatic response selection process for the left-hand (upper vs. lower button), the uncertainty in this decision should have been the *same* for all distractors L, P, and H. In turn, any effect of uncertainty (P vs. L/H) cannot be explained just by a motor component. After the 2-s search interval, the reaction time was displayed inside the shape target if the right-hand response was correct. An “F” (for false) was displayed for an incorrect response. The next trial started after a random pause (empty screen) of 1–3 s.

Written instruction about the search task were presented prior to the experiment. Participants were informed: (1) that all search trials began with a gray fixation cross; (2) that they should find and fixate the diamond shape as quickly as possible in order to identify the line orientation within the diamond; (3) that the color distractor should be ignored; (4) that the feedback screen would show their reaction time; (5) that they should try to respond as quickly as possible; and (6) that there would be no monetary reward in search trials.

#### Trial Sequence

Across learning trials, each of the six color cues was displayed at each of the six search array positions. Relative to the color cue, the white distractor was shown at one of the four adjacent positions (but never at the exact opposite position). Accordingly, the white distractor was presented an equal number of times in the same hemifield or the opposite hemifield as the color cue. The combination of color, cue position, and distractor position resulted in 6 × 6 × 4 = 144 trials in total. Training trials were presented in six successive blocks of 24 trials with four replications per color. Each participant received a different pseudo-random sequence of trials with the restriction that the same type of reinforcement (L, P, H) did not occur more than three times in a row.

The positioning of the shape target and the color distracter was similar to the learning task. Across trials, the shape target was presented: (a) at all six positions; (b) in the context of each of the three color distractors; (c) at each of the four positions adjacent to the target (but never at the exact opposite position). The combination of target position and distractor position resulted in 6 × 4 = 24 trials that we replicated twice to yield 48 search trials per distractor color. Additionally, the experiment included 48 no-distractor baseline trials which presented only the shape target yielding a total of 4 × 48 = 192 search trials. These were randomly interspersed with learning trials from Block 3 to Block 6 of training where each block contained 24 learning trials (4 replications of each of 6 color cues) and 48 search trials (12 replication of each of three color distractors plus 12 baseline trials). Each participant received a different pseudo-random sequence of trials with the restriction that the same color did not occur more than three times in a row. With 144 learning trials, and 192 search trials, the experiment consisted of 336 trials in total.

### Dependent Variables and Data Analysis

Custom MATLAB (The MathWorks, Inc., 2012) software was used for the signal conditioning of eye position traces and the detection (velocity-based algorithm) and parametrization of ocular fixations (Koenig, 2010; Koenig and Lachnit, 2011). In the learning task, fixations were classified as fixations on the color cue, the white distractor, or on one of the remaining gray distractors. In the search task, fixations were classified as directed to the shape target, the color distractor, or one of the gray distractors. In the circular search array these stimuli were positioned at a radius of *r*_stim_ = 100 mm and polar angles of φ = 0°, 60°, 120°, 180°, 240°, and 300° respectively. A fixation was assigned to one of these stimuli if its angular deviation from the stimulus direction was less than 30° and its distance from central fixation exceeded 50 mm. For learning trials, the duration of all fixations on the color cue that occurred before participants made the left-hand choice response was summed to yield the total dwell time on the color cue per trial. For search trials, the first fixation within each trial was identified and we scored: (a) whether this fixation was on the target or the distractor; (b) the latency of this first fixation; and (c) the duration of this fixation. Based on these scores, we analyzed three dependent variables in the search task: *Capture frequency* was computed as the frequency of trials in which the first fixation was on the color distractor. In these capture trials, participants had to disengage from the color distractor in order to look at the shape target eventually and we analyzed *capture duration* as the dwell time on the distractor. Finally, in non-capture trials (first fixation on shape target), we expected the exact onset latency of the target fixation to be dependent on how much time it took to successfully suppress the distractor. We analyzed *suppression duration* as the target fixation latency in non-capture trials. Latencies and durations were log-transformed to achieve normality for statistical inference and model predictions. Statistical plots are based on back-transformed values to be more descriptive.

The analysis included valid trials only. A trial was excluded if participants did not fixate on the central fixation cross when the search display appeared (8%) or did not move their eyes to the target within the 2 s of search array presentation (6%). Also, a trial was excluded if the search interval included any signal loss (mostly due to blinks) that could have masked a relevant fixation (12%). With these criteria, 74% of the search trials were regarded as valid and taken into account for further analysis.

We used (generalized) linear mixed models (G)LMMs to analyze the data (Bates et al., 2015). This approach allowed the modeling of binary outcome variables (0, 1) such as the incidence of distractor fixations per trial to yield valid probability estimates using a logistic link function. Also, the approach allowed for the modeling of unbalanced data sets for example when the duration of distractor fixations could only be estimated from participants and trials where such distractor fixations actually occurred. Analysis of variance (ANOVA) were computed from the mixed-models which included random effects to account for repeated measures. Significance tests for model terms were derived from the comparison of the full model including the respective term with a reduced model dropping the term. For logistic GLMMs this statistical inference was based on likelihood-ratio tests. For normal LMMs, *F*- and *t*-statistics were computed from approximate degrees of freedom (df) using the method of Kenward and Roger (1997) and (Halekoh and Højsgaard, 2014). As measures of effect size, we report the standardized regression weights (beta) for single predictors (contrasts, continuous predictors) and a *η*^2^-statistic for model terms that include multiple predictors (multilevel factors). The latter statistic was computed as the squared bivariate correlation between the observed values and the predicted values by that factor. Unless stated otherwise, models included maximal random-effects structures to allow for confirmatory testing (Barr et al., 2013). All statistical analyses were conducted using the R language and environment for statistical computing (R Core Team, 2016).

## Results

### Learning Task

#### Accuracy

**Figure 4:**
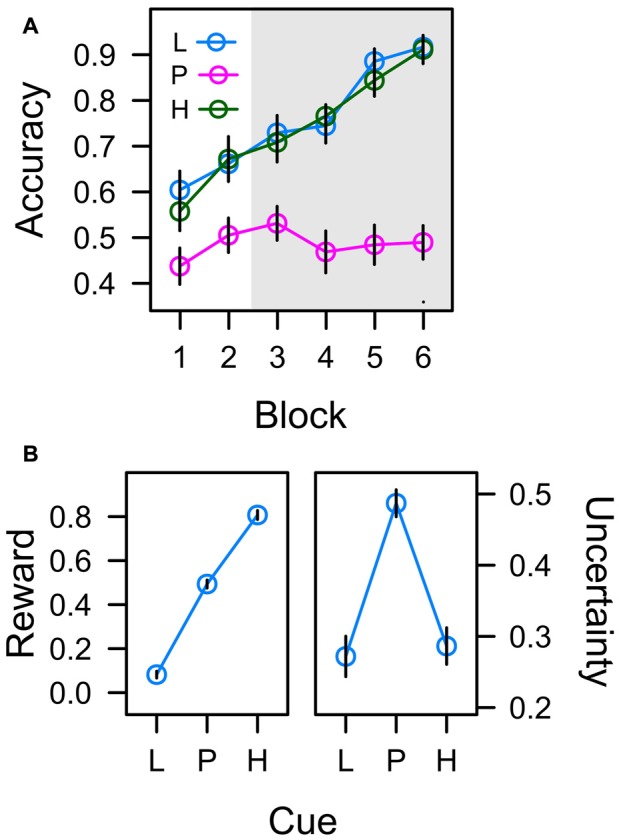
**(A)** Frequency of correct responses to low reward cues (L; collapsed across trained colors L1 and L2), partially reinforced cues (P; collapsed across P1 and P2), and high reward cues (H; collapsed across H1 and H2) in six successive blocks of learning trials. The gray shaded area marks the time when learning trials were randomly interspersed with search trials. **(B)** Average values associated with cues L, P, and H in the last four blocks of training. Reward expectancy is computed as the average amount of monetary reward earned in learning trials (as a fraction of 10 cent). Uncertainty (right panel) is computed as the standard deviation of correct responding. Error bars depict the standard error of the mean.

In the learning task, participants had to acquire associations between six different color cues and a left-hand manual choice response (upper vs. lower button) that resulted in either low reward (1 Cent) or high reward (10 Cent) if correct. Figure 4A depicts the accuracy of this manual response over the course of six successive blocks of learning. For the low reward color cue (L) and the high reward color cue (H), the correct response was predictable and accuracy thus continuously increased from about 60% to 90% as participants learned the correct color-response mapping. In contrast, for the color cues in the uncertain partial reward condition, one of the two response alternatives was randomly reinforced from trial to trial, and in turn the accuracy of this unpredictable response was at chance level throughout the experiment.

An ANOVA with factors cue (L, P, H) and block (1–6) revealed significant effects of cue, *F*_(2,45)_ = 34.004, *p* < 0.001, *η*^2^ = 0.286, and block, *F*_(4,93)_ = 25.299, *p* < 0.001, *η*^2^ = 0.168, that were modulated by a Cue × Block interaction, *F*_(7,155)_ = 5.420, *p* < 0.001, *η*^2^ = 0.083. A significant contrast for factor cue with weights λ_[L, P, H]_ = [0.5, −1, 0.5], *t*_(46)_ = 8.237, *p* < 0.001, *β* = 0.771, confirmed that responding in the predictive conditions L and H was more accurate than in the uncertain condition P. There were no differences in accuracy between predictive conditions L and H in any block of training (all *t* < 1).

#### Reward

If participants responded correctly, they earned 1 Cent in the low reward condition, and 10 Cent in the partial and high reward conditions in order to establish reward expectancies of L (1 Cent) < P (5 Cent) < H (10 Cent). The left panel of Figure 4B depicts the average amount of monetary reward (as a fraction of 10 Cent) earned in experimental conditions L, P, and H during blocks 3–6 i.e., during the time when search trials were interspersed with learning trials. A linear trend was highly significant, *t*_(23)_ = 25.690, *p* < 0.001, *β* = 0.886. With respect to attentional measures reported below, if associative learning established an* attentional bias for reward*, oculomotor measures of this attention bias should accord to the pattern L < P < H.

#### Uncertainty

Cues in the learning task were associated with different degrees of uncertainty. Whereas the correct manual response (and in turn the amount of reward) was predictable in experimental conditions L and H, the manual response was unpredictable in condition P inducing high uncertainty. The right panel in Figure 4B depicts the actual uncertainties associated with the color cues (plotted as the standard deviation of correct responding). A quadratic contrast was highly significant with *t*_(23)_ = −7.529, *p* < 0.001, *β* = −0.238. If associative learning established an *attentional bias for uncertainty*, oculomotor measures of this attention bias reported below should accord to this pattern L < P > H.

#### Manual Response Latency

**Figure 5:**
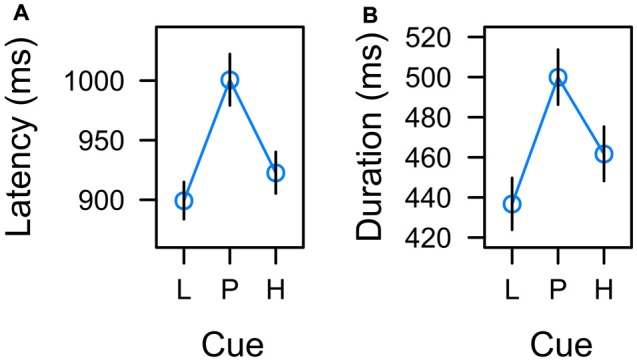
Effects of low reward (L), partial reinforcement (P), and high reward (H) on **(A)** the latency of the manual response and **(B)** the duration of fixations on the color cue in the learning task. Graphs depict back-transformed values from the log-scale used for statistical inference. Error bars depict the standard error of the mean.

In each learning trial the manual response for reward was valid during the entire 2-s interval during which the color cue was presented. We analyzed the manual reaction time in the learning task as a measure of the total decision time associated with predictive vs. uncertain cues. A mixed-effects ANOVA indicated that the cue factor had a significant effect on response latency, *F*_(2,22)_ = 5.508, *p* = 0.011, *η*^2^ = 0.043. Inspection of Figure 5A suggests that the effect of cue was an effect of uncertainty with longest response latencies for the uncertain cue.

To further confirm that response latency was linked to uncertainty rather than expectancy, we adopted a model comparison perspective. The *F*-test for an overall effect of cue reported above was based on a LMM including a set of two orthogonal contrasts. One contrast coded for a linear trend caused by reward expectancy, λ_[L, P, H]_ = [−1, 0, 1]. The other contrast coded for a quadratic trend caused by associative uncertainty, λ_[L, P, H]_ = [−0.5, 1, −0.5]. Both contrasts were standardized for comparability yielding λ_[L, P, H]_ = [−1.225, 0, 1.225] and λ_[L, P, H]_ = [−0.707, 1.414, −0.707] respectively. The full model included both variables, reward expectancy and uncertainty, to predict response latency and we tested whether dropping either predictor would significantly deteriorate the goodness of fit. The first row of Table 1 (*Manual response latency*) presents the results. While a model dropping reward expectancy (and including uncertainty only) was as good as the full model, dropping uncertainty from the full model significantly deteriorated the goodness of fit. In sum, these tests confirmed that manual response latency was linked to uncertainty rather than expectancy.

**Table 1:** Model comparisons for the inclusion of expectancy and uncertainty as predictors of attentional bias during learning and search.

		Full model	Expectancy	Uncertainty
**Learning task**				
*Manual response*	*AIC*	−227.250	−228.289	−220.753
*latency*	*t (23)*		0.961	3.122
	*p*		0.342	0.004**
	β		0.049	0.207
*Total dwell time*	*AIC*	2133.812	2133.571	2138.096
	*t(22)*		1.319	2.589
	*p*		0.200	0.016*
	β		0.046	0.129
**Search task**				
*Distractor fixation*	*AIC*	2903.175	2912.593	2901.178
*frequency (capture frequency)*	*χ*^2^(1)		11.418	0.003
	*p*		0.001***	0.956
	β		0.057	−0.004
*Target fixation*	*AIC*	24.226	24.592	27.292
*latency (suppression duration)*	*t(22)*		1.532	2.247
	*p*		0.139	0.034*
	β		0.063	0.090
*Distractor fixation*	*AIC*	237.360	235.557	242.690
*duration (capture duration)*	*t(20)*		0.437	2.878
	*p*		0.666	0.009**
	β		0.024	0.182

Note. The expectancy contrast codes for an increase L < P < H in attentional with coefficients −1, 0, 1. The uncertainty contrast codes for the pattern L < P > H with coefficients −0.5, 1, −0.5. Both contrast were standardized for comparability yielding λ_[L, P, H]_ = [−1.225, 0, 1.225] and λ_[L, P, H]_ = [−0.707, 1.414, −0.707], respectively. Goodness of fit is given as the Akaike information criterion (AIC). Statistical tests (t, p) refer to the comparison of the full model including both predictors expectancy and uncertainty with a reduced model dropping either expectancy or uncertainty. Effect sizes (β) refer to the standardized regression weights of the contrast. *p < 0.05, **p < 0.01, ***p < 0.001.

#### Total Dwell Time

In accord with previous experiments (Hogarth et al., 2008; Beesley et al., 2015), we analyzed the total dwell time on the color cues before participants pressed one of the response buttons. A mixed-model ANOVA revealed a significant main effect of cue, *F*_(2,21)_ = 3.604, *p* = 0.045, *η*^2^ = 0.019. As depicted in Figure 5B, the effect of cue again appeared to correspond to uncertainty with longer fixations on uncertain cues P than predictive cues L and H. Table 1 (*Total dwell time*) depicts model comparisons following the same rationale as detailed in the preceding paragraph. While a model dropping reward expectancy (and including uncertainty only) was as good as the full model, dropping uncertainty from the full model significantly deteriorated the goodness of fit. Again, these tests confirmed that total dwell time on the cues was linked to their uncertainty rather than to the amount of associated reward. However, the time participants spent fixating the color cues were strongly related to the manual reaction times. On a single trial level, mixed-model regression revealed, that the latency of the participants manual response predicted fixation duration with *t*_(22)_ = 8.964, *p* < 0.001, *β* = 0.516 (both measures *z*-standardized). This correspondence was due to the fact that participants had no reason to look anywhere else than at the color cue up to the time they committed to a response. We computed an analysis of covariance to examine if there was any residual effect of the cue factor on fixation duration after controlling for this dependency. The analysis revealed no residual effect of cue, *F* < 1, indicating that total fixation duration in the learning task was strongly confounded with the requirement of choosing a manual response.

### Search Task

In the search task trained colors were introduced as irrelevant distractor during visual search for a shape target, and we measured the potential of valued color distractors to capture and hold overt attention.

#### Capture Frequency

**Figure 6:**
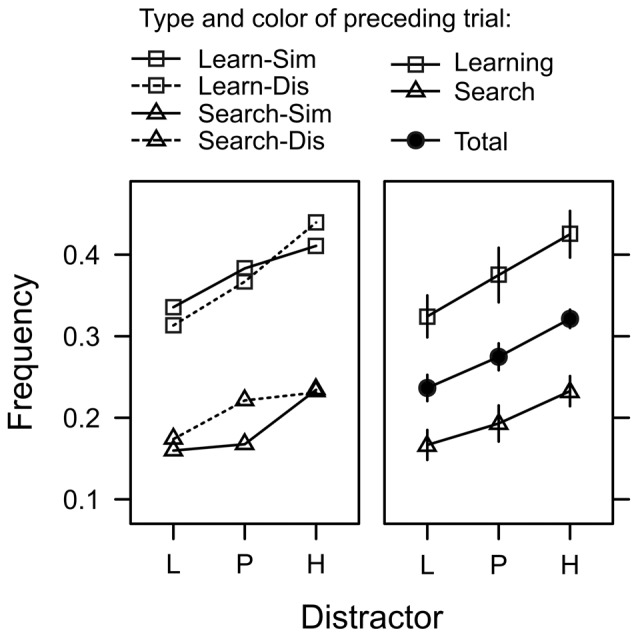
Frequency of trials in which the first fixation was captured by the color distractor during search trials. Distractors were associated with low reward (L), partial reinforcement (P), and high reward (H). The left panel depicts four different cases depending on whether the preceding trial was a learning trial or a search trial (Learn/Search), and whether the color in the preceding trial was similar or dissimilar (Sim/Dis). The right panel shows the same data collapsed across color similarity (which exhibited no significant effect) and also shows the main effect of distractor collapsed across all preceding trial effects (solid circles). Relative frequencies are back-transformed from the logit-scale of the generalized mixed model. Error bars indicate the standard error of the mean.

In 56% of the trials, the first fixation was on the shape target, while the color distractor captured gaze in about 28% of the trials. In the remaining 16% of the trials the participants first fixation was on one of the gray distractors. We used a generalized-mixed effects model (GLMM) to analyze the incidence of attentional capture by the valued color distractors. For each search trial featuring a color distractor, we scored whether the first peripheral fixation was on the color distractor or not (represented by values of 1 or 0 respectively) and used a logistic link function to model this binary outcome variable. Random intercepts allowed for random variation between subjects. Our main interest was whether capture frequency would be influenced by the learned value of the distractor (L, P, H). However, the model also included factors to explore effects of inter-trial priming and task-switching. Since search trials and learning trials were randomly intermixed, for each search trial we registered whether the directly preceding trial was also a search trial or a learning trial (factor *preceding type*), Furthermore, irrespective of the preceding trial type, the color in trial *n* − 1 could have been either similar or dissimilar to the color in trial *n* (factor *preceding color*). Lastly, the model included the log-latency of the first fixation as a continuous predictor. Likelihood ratio test for the inclusion of these factors in the model revealed a significant effect of fixation latency, $\chi_{\left(2\right)}^{2}$ = 8.569, *p* = 0.013, *β* = 0.302, that will be explored further down below. There was a significant main effect of distractor, $\chi_{\left(2\right)}^{2}$ = 8.569, *p* = 0.013, *η*^2^ = 0.003, and preceding type, $\chi_{\left(1\right)}^{2}$ = 55.981, *p* < 0.001, *η*^2^ = 0.015, but no effect of preceding color, $\chi_{\left(1\right)}^{2}$ = 0.269, *p* = 0.603, *η*^2^ < 0.001 and no interactions (all *χ*^2^ < 1, all *p* > 0.50). As evident from Figure 6, the main effect of preceding type exhibited the typical characteristics of a task-switching effect. The frequency of attentional capture by the color distractor was higher on switch trials (where trial *n*−1 was a learning trial) than on repetition trials (where trial *n*−1 was a search trial) indicating that participants were better prepared to suppress the distractor on repetition trials. However, as can be seen in Figure 6 this task-switching effect did not modulate the main effect of distractor value, which was caused by a linear trend L (1 Cent) < P (5 Cent) < H (10 Cent) in both, repetition trials, $\chi_{\left(1\right)}^{2}$ = 6.730, *p* = 0.009, *β* = 0.045, and switch trials, $\chi_{\left(1\right)}^{2}$ = 5.453, *p* = 0.019, *β* = 0.076.

To further confirm that capture frequency was linked to reward expectancy rather than uncertainty, we fitted a generalized mixed model that included the expectancy and uncertainty contrasts as detailed in Table 1 (*Distractor fixation frequency*). The full model included both variables, reward expectancy and uncertainty, to predict capture frequency. Dropping reward expectancy from the full model significantly deteriorated the goodness of fit while a model dropping uncertainty (and including expectancy only) was as good as the full model. These tests confirmed that capture frequency was linked to reward expectancy rather than uncertainty.

#### Fixation Latency

**Figure 7:**
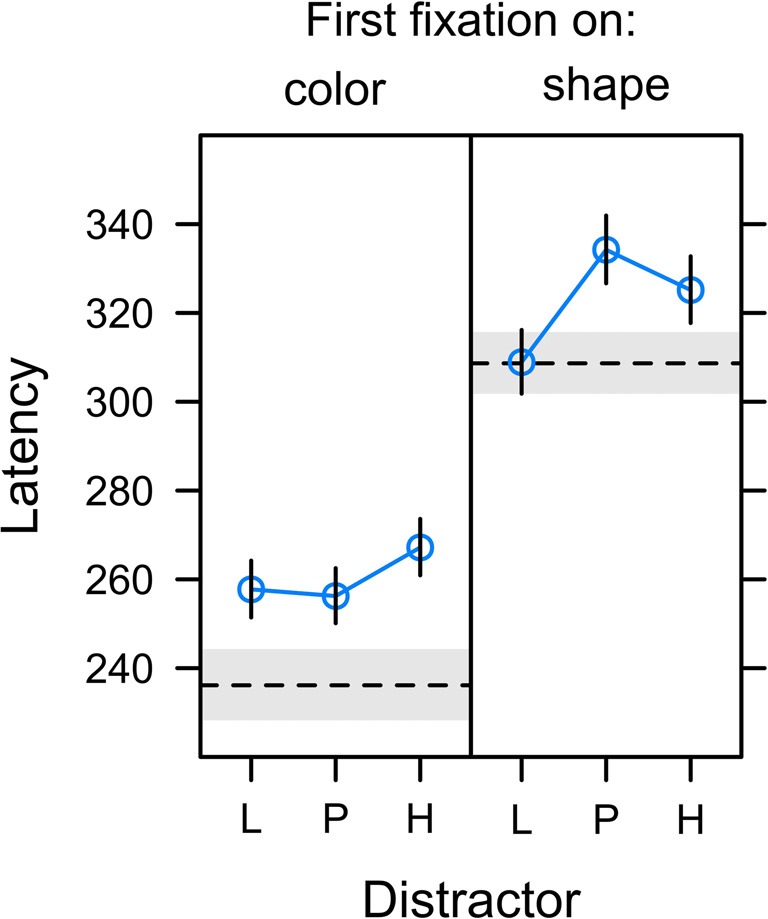
Latency of first fixations relative to the onset of the search display in trials in which the first fixation was on the color distractor (left panel; capture trials) or the shape target (right panel; non-capture trials). Distractors were associated with low reward (L), partial reinforcement (P), and high reward (H). The dashed horizontal lines depict the latency of fixations on gray distractor (left panel) and the latency of target fixations in the no-distractor baseline condition (right panel). Graphs depict back-transformed values from the log-scale used for statistical inference. Error bars and gray error band depict the standard error of the mean.

Figure 7 depicts the onset latency of the first fixation in a search trial depending on whether this fixation was on the color distractor (left panel) or the shape target (right panel). A comparison between panels reveals that fixations on the color distractor started earlier after onset of the search display than fixations on the shape target, *t*_(22)_ = −6.971, *p* < 0.001, *β* = −0.375. This explains the effect of fixation latency on capture frequency as reported in the preceding section and indicates that a successful suppression of the distractor (first fixation on shape target in non-capture trials) was more likely for long latency fixations allowing more time for the target selection processes. In contrast, it was the characteristic of erroneous distractor fixations (first fixation on the color distractor in capture trials) to be of short latency.

The analysis of distractor fixation latencies (left panel) revealed no differences between distractor values L, P, and H, *F*_(2,20)_ = 1.144, *p* = 0.338, *η*^2^ = 0.006. For target fixations (right panel), the omnibus *F*-test fell just short of statistical significance, *F*_(2,21)_ = 3.443, *p* = 0.051, *η*^2^ = 0.014. However, there was a significant effect for the specific contribution of uncertainty as shown in Table 1. Again, we compared the fit of the full model including both expectancy and uncertainty to a reduced model dropping either of these contrasts. While a reduced model dropping reward expectancy was as good as the full model, dropping uncertainty from the full model significantly deteriorated the goodness of fit. These tests confirmed that the latency of fixations on the shape target was linked to the uncertainty associated with the competing color distractor rather than the amount of reward associated with the distractor.

#### Capture Duration

**Figure 8:**
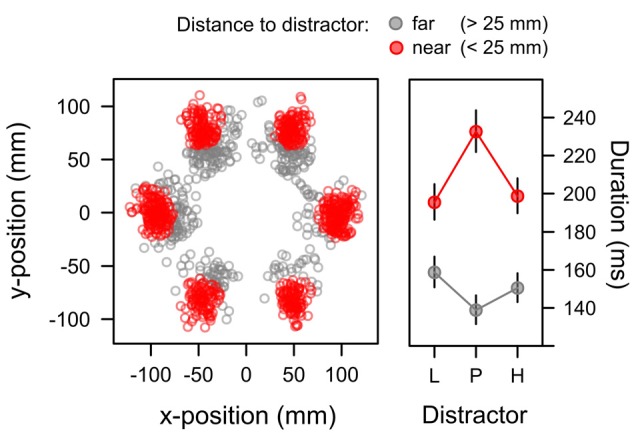
Position (left panel) and duration (right panel) of distractor fixations in the search task. Fixations landing on the distractor (red; <25 mm from the distractor position) exhibited durations that were linked to the associative uncertainty of the distractor with longer durations for the high-uncertainty distractor P than for the low-uncertainty distractors L and H. Fixations with a greater distance to the target mostly showed an undershoot (when the first saccade was too short to reach distractor) and exhibited the inverse pattern of fixation durations. Fixations on gray distractors (not shown in the Figure) had durations of 136 ms (*SE* = 10.36) and 107 ms (*SE* = 9.04) in the near and far condition respectively. The right panel depicts back-transformed values from the log-scale. Error-bars depict the standard error of the mean.

For capture trials in which participants first attended to the irrelevant distractor, we analyzed the duration of these distractor fixations as a third measure of attentional bias. Initial data exploration showed that beyond the learned distractor value fixation duration was also dependent on the exact landing position of the fixation relative to the distractor. Figure 8 highlights this dependency. The scatterplot in the left panel depicts the positions of distractor fixations in the search task where the distractor appeared with equal probability at each of the six positions in the search array. The distance of the fixation from the exact distractor position is color-coded within the panel. Red dots mark the occurrence of fixations that were near the distractor (not more than 25 mm from the center of the distractor). In contrast, gray dots mark the occurrence of fixations that were further away (more than 25 mm). The scatterplot in Figure 8 suggests that this latter subset of far fixations mostly resulted from an undershoot where the first eye movement to the periphery was too small to reach the exact distractor position (and a second eye movement might have been required to reach the distractor). The right panel depicts the duration of both types of fixations (near, far) for each distractor value (L, P, H). Marginal means were computed from a LMM that included random intercepts and slopes within participants to account for repeated measures. In this model a significant main effect of distance, *F*_(1,21)_ = 36.663, *p* < 0.001, *η*^2^ = 0.124, was caused by the longer duration of near fixations, *M* = 208 ms, *SE* = 5.655, than far fixations, *M* = 149 ms, *SE* = 4.461. A main effect of distractor value was not significant, *F* < 1, however, there was a significant Distractor × Distance interaction, *F*_(2,19)_ = 5.327, *p* = 0.014, *η*^2^ = 0.017. The interaction was caused by an inverse data pattern with *longer* dwell times on the uncertain, partially reinforced distractor for near fixations as indicated by a significant quadratic contrast λ_[L, P, H]_ = [−0.5, 1, −0.5], *t*_(18)_ = 2.912, *p* = 0.009, *β* = 0.187, while the inverse pattern for far fixation (with shorter dwell time on uncertain cues) did not reach the level of statistical significance, *t*_(19)_ = −1.711, *p* = 0.104, *β* = −0.122.

Table 1 depicts the model comparisons for the importance of expectancy and uncertainty in predicting the duration of fixations on the distractor (near fixations). While dropping uncertainty from the full model significantly deteriorated the model fit, a model dropping expectancy (and including uncertainty only) was as good as the full model. These tests confirmed, that capture duration was linked to uncertainty rather than reward expectancy.

#### Manual Response

**Figure 9:**
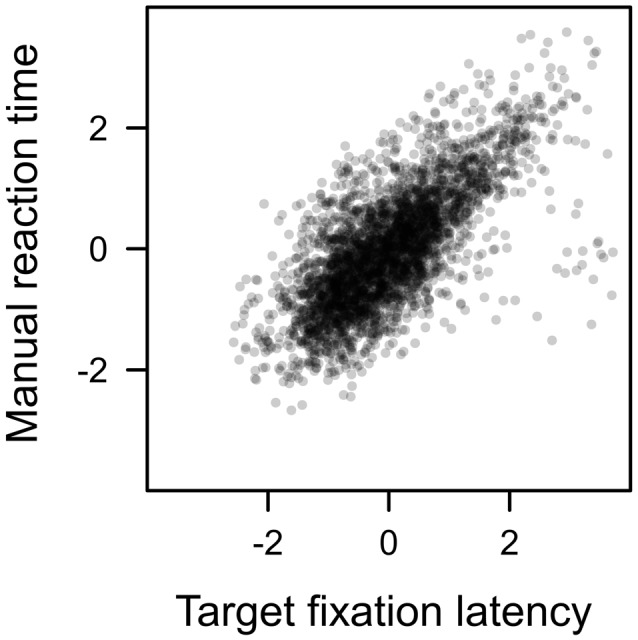
Correlation between manual reaction time and target fixation latency in the search. Both measures standardized (*r* = 0.701).

Each search trial required a manual response to the target. This response was correct in about 94% of the trials with no difference between distractor types, *F* < 1. Since a correct response *required* the fixation of the shape target, we expected the latency of that manual response to be highly dependent on the eye movement pattern performed before the target was fixated. For example, if participants first fixated on the color distractor, the latency of the first target fixation of course was prolonged by the duration of the distractor fixation. Figure 9 plots the manual reaction time against the latency of the first target fixation (both measures z-standardized). A linear effect of target fixation latency was highly significant, *t*_(23)_ = 14.670, *p* < 0.001, *β* = 0.701. The later the eyes landed on the shape target in search trials, the later participants were able to identify the line orientation and perform a manual response. An analysis of covariance revealed no residual effect of distractor value on manual reaction times after controlling for this dependency, *F* < 1.

## Discussion

### Expectancy Bias

In accord with previous studies (Anderson et al., 2011a,b; Anderson and Yantis, 2012; Theeuwes and Belopolsky, 2012; Le Pelley et al., 2015), we found evidence of attentional capture by reward-associated stimuli. In the search task of our experiment that introduced reward-associated colors as task-irrelevant distractors, the *frequency of attentional capture* by these distractors was linked to reward expectancy: after onset of the search display, the probability of looking at the color distractor first (before looking at the shape target) was higher for high reward distractors than for low reward distractors with an intermediate capture rate for the partially reinforced distractor. This pattern supports the idea that reward expectancy was actually represented as the product of reward value and probability. The partially reinforced distractor (P), followed by 10 Cent in 50% of the learning trials, thus earned 5 Cent on average and elicited capture at an intermediate rate in between the low reward distractor (L; 1 Cent) and the high reward distractor (H; 10 Cent).

In contrast to previous studies that used a blocked presentation of search trials after an initial continuous block of learning trials (Anderson et al., 2011a,b; Anderson and Yantis, 2012), the current experiment used an intermixed procedure in which learning trials and search trials were presented in a random sequence while the type of the initial fixation cross functioned as a task cue that instructed participants about the type of the impending trial. We used the intermixed procedure to prevent continuous extinction learning that would have resulted from a blocked presentation of non-reinforced search trials. As outlined in the introduction, during such a continuous extinction block the reward associations of the distractors form the same rank order as the associated prediction errors (uncertainties). If a high reward distractor captures attention during such blocked extinction it is unclear whether this capture occurs because of the cues residual association with reward (expectancy) or because of the fact that a high-expectancy cue under extinction also causes a high prediction error (uncertainty). In contrast to the blocked design, our intermixed procedure allowed to attribute the observed pattern of capture frequency L < P < H to the distractors association with reward.

However, the intermixed procedure introduced the possibility that capture by the reward-associated distractors in search trials was not “automatic” but rather stemmed from a task-switching effect where the task-set for the learning task on trial *n* − 1 could have bled into the search task on trial *n* favoring the selection of the color distractor because of its residual designation as a task-relevant target. Residual costs for such a task set reconfiguration (TSR; Monsell, 2003) have been reported for task-switching experiments that allowed preparation times of 5 s or more (Kimberg et al., 2000; Sohn et al., 2000) well in excess of the 2-s task cue in the current experiment. In accord with such an interpretation, our data showed a clear task switching effect where the probability of attentional capture by the color distractor in search trial *n* was higher on switch trials (*n − 1 learning* trial) than on repetition trials (*n* − 1 search trial; see Figure 6). The effect is readily explained by a learning task set “attend to color” that still was active on search trials. However, our analysis also revealed that the task switching effect did *not* modulate the observed expectancy effect L < P < H which was present on switch trials as well as repetition trials. In other words, when the search display appeared in repetition search trials, participants were better prepared to ignore the color distractor in general but they were *not* better prepared to ignore the specific reward value of the color. From our perspective, this suggests that the reward value of the color distractor in the search task was retrieved automatically at least to some extent.

Our demonstration of attentional capture by reward-associated but task-irrelevant stimuli outside the original acquisition context adds to a growing body of empirical research about the effects of reward on attention (Anderson et al., 2011a,b; Anderson and Yantis, 2012; Awh et al., 2012; Theeuwes and Belopolsky, 2012; Le Pelley et al., 2015). However, an additional new finding of the present experiment was that some measures of overt attention in the search task were linked to uncertainty rather than reward with a strong bias for distractors that consistently caused a high prediction error in the learning task.

### Uncertainty Bias

Evidence for an uncertainty-linked bias was evident for the *target fixation latency* in non-capture trials as well as the *distractor fixation duration* in capture trials. In non-capture trials, participants successfully suppressed the color distractor and directed their first fixation to the shape target. Relative to the no-distractor baseline condition, the latency of the first target fixation was increased when an uncertain distractor was present in the search display. One possible interpretation of this effect is that it was linked to the time required to suppress the distractor to an extent that allowed the selection of the shape target.

In capture trials, the duration of fixations on the distractor was linked to uncertainty as well. The time participants fixated on the distractor before they moved their eyes to the shape target was longer for partially reinforced, uncertain cues of reward (P) than reliable cues of either low reward (L) or high reward (H). This distractor effect L < P > H in the search task mimicked the prolonged dwell time on uncertain cues during learning trials (also see Hogarth et al., 2008; Beesley et al., 2015). However, longer fixations on uncertain cues during learning were confounded with the longer latency of the manual response to these uncertain cues that was *required* in the learning task. Statistically, there was a strong correlation between the two measures in the learning task and there was no residual effect of uncertainty on dwell time after controlling for that dependency. Whereas the longer dwell on uncertain cues in the learning task thus could have been caused by the task requirements, the longer capture duration of uncertain distractors in the search task was evident without the requirement to predict an outcome associated with the distractor. In fact, the distractor could have been completely ignored in the search task. If participants fixated on the distractor in search trials because of some residual activation of the learning task set, the TSR that eventually led to the fixation of the shape target took longer if the fixation was on an uncertain distractor associated with a high prediction error in the search task. From our perspective, the finding yields some support for the idea that associative uncertainty automatically prolonged attentional holding.

Please also keep in mind that the observed uncertainty bias in the search task cannot be attributed to some automatic response preparation process that transferred from the learning task to the search task. The experiment was specifically designed to match the predictiveness of the distractor colors in the search task for the left-hand response from the learning task. For example, when the high-reward distractor was presented during search, it was equally similar to two high-reward cues from the learning tasks that were trained with different response buttons. With respect to predicting whether a distractor color was associated with either the upper or lower left-hand button *all* distractors low, partial, and high exhibited the *same* amount of uncertainty. By exclusion, we would argue that the bias for the uncertain distractor in the search task existed because associative learning automatically increased the cue’s bias weight every time it lead to a high prediction error in the learning task. This idea is at the heart of several attentional learning theories (Pearce and Hall, 1980; Pearce et al., 1982; Pearce and Mackintosh, 2010; George and Pearce, 2012; also see Le Pelley, 2004). Our finding of an uncertainty-linked attention bias *outside* the original learning context to our knowledge has not been reported previously and provides further support for the theoretical idea that learners pay more attention to cues that consistently have produced a high prediction error in the past.

Our discussion of distractor fixation duration above has focused on fixations that were *on* the distractor. As detailed in the “Materials and Methods” Section, distractor annuli measured 31 mm in diameter and around the distractor position we defined a circular area of interest that was 5 cm in diameter to classify fixations as “on the distractor” (referred to as *near fixations* in the results section). Fixations that landed outside this area of interest (*far fixation*) mostly were due to an undershoot where the eye movement starting from central fixation did not quite reach the distractor position. Although a distractor effect on the duration of these far fixations failed to reach statistical significance, at least descriptively it revealed an inverse pattern of durations compared with near fixations (see Figure 8). We would like to point out that this observation also is consistent with a bias for high-uncertainty distractors. If a high-uncertainty distractor exhibits a high attentional bias weight, a (near) fixation that lands on the distractor would be prolonged but a (far) fixation landing at a distance that would require a second corrective saccade to finally land on the distractor should be only brief in order to reach the high-value distractor as quickly as possible. Our experiment was not specifically designed to examine this effect but future research should try to substantiate this hypothesis.

### Sequential Processing of Expectancy and Uncertainty?

**Figure 10:**
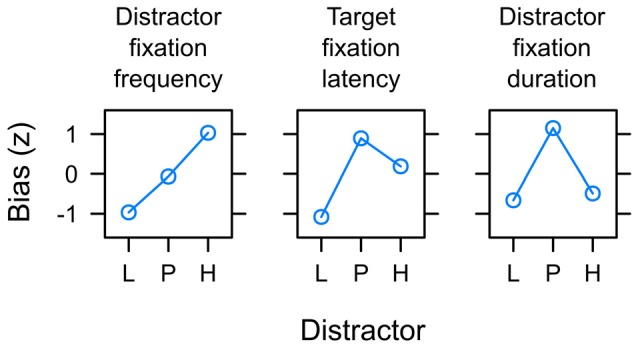
Summary of main empirical findings with respect to the potential of valued distractors to capture and hold attention in the search task. Distractors were associated with low reward (L), partial reinforcement (P) and high reward (H) respectively. The y-axis depict the standardized attentional bias observed with respect to the frequency of capture by the valued distractor (left panel; see Figure 6), the latency of target fixations in the presence of the valued distractor (mid panel; see Figure 7), and the duration of distractor fixations in capture trials (right; see Figure 8). Distractor fixations in capture trials started around 260 ms after onset of the search display. Target fixations in non-capture trials started around 320 ms on average. With an average duration of 210 ms, distractor fixations in capture trials ended around 470 ms. From left to right the differences between measures thus may constitute a gradual shift from an initial expectancy bias L < P < H to a subsequent uncertainty bias L < P > H.

Figure 10 summarizes the main empirical finding as discussed above. Whereas capture frequency was linked to reward expectancy (left panel), target fixation latency (mid panel) and capture duration (right panel) were rather linked to uncertainty. One possible perspective on this dissociation between different measures is to acknowledge that these were measures of attentional bias at different times during the search interval. The oculomotor decision to select the distractor for the first fixation appeared early after the onset of the search display at about 260 ms on average (see left panel of Figure 7). The frequency of distractor fixations as shown in the left panel of Figure 10 thus was a measure that was sensitive to an early influence of the distractor on attentional selection. In contrast, if participants successfully suppressed the distractor and instead looked at the shape target this fixation started around 320 ms after onset of the search display (see right panel of Figure 7). The target fixation latency in the presence of a competing color distractor as shown in the mid panel of Figure 10 thus was a measure that was sensitive to a later influence of the distractor on attentional selection. Finally, with an average duration of fixations on the distractor of about 210 ms (see Figure 8) the duration was determined by the disengagement from the distractor around 470 ms. With these different times of origin, one might arrive at the assumption that the three measures might in fact provide insight into how the representations of reward and uncertainty changed during the search interval. From this perspective, the different biases shown in Figure 10 from left to right in fact could show the within-trial transition of an early bias for reward to a later bias for uncertainty. In accord with the suggested temporal dynamics, a pattern of reward-related capture *frequency* and a stronger influence of uncertainty on capture *duration* has been reported for distractors previously associated with aversive electric shock in human fear conditioning (Koenig et al., 2017) and for reward-associated distractors that *always* were task-irrelevant (Koenig et al., in preparation).

Furthermore, the suggested temporal dissociation of reward and uncertainty is in accord with empirical evidence about the response of dopamine neurons in the midbrain and striatum. Two different perspectives suggest that dopamine signaling plays an important role in value-based attention. On the one hand, there now is substantial empirical evidence that the responses of dopamine neurons comply with the basic predictions of associative learning theories (Waelti et al., 2001) and code for prediction errors, reward value, and reward uncertainty (Fiorillo et al., 2003; Schultz et al., 2008; Schultz, 2016). On the other hand, dopamine signaling has been explicitly linked to the representation of the *motivational salience* (Schultz, 2016) of stimuli associated with reward (Hickey and Peelen, 2015). In particular, Anderson et al. (2016) reported that the potential of previously rewarded distractors to automatically capture attention during visual search was correlated with changes in available D_2_/D_3_ dopamine receptors. Importantly, the response of dopamine neurons has been described as bi-phasic with a first fast component that linearly increases with the expected amount (or probability) of reward and a second slow component that gradually builds up to represent reward uncertainty (Fiorillo et al., 2003; Schultz, 2016). As discussed above, the patterns of observed attention bias in our search task could have been caused by such representational dynamics. Future research should try to refine methods to examine the possible transition of attentional biases within trial. For example, for an experimental manipulation of distractor fixation latencies in capture trials (e.g., in a gap-overlap design), we would expect late onset distractor fixations to be more strongly affected by uncertainty than early onset distractor fixations. At any rate, the question if associative learning establishes a specific attentional bias may by wrong at the expense of neglecting the possibility that the brain may hold multiple biases at once.

## Conclusion

As recently criticized by Anderson (2015) “current theoretical accounts of value-driven attention […] are largely limited to a description of the phenomenon—that reward history plays a direct role in the control of attention”. Associative learning theories may provide one “missing link” to theories of how the brain ascribes value to external stimuli. Our experiment revealed that associative learning established an attention bias that corresponded to the amount of expected reward as well as associative uncertainty. These biases may be characterized by different onset latencies with reward value being represented with a lower latency than uncertainty.

## Author Contributions

SK conducted the experiment, analyzed the data and drafted the manuscript. HK, MU, AS and HL made contribtions to the design of the experiment and helped revising the manuscript.

## Conflict of Interest Statement

The authors declare that the research was conducted in the absence of any commercial or financial relationships that could be construed as a potential conflict of interest.

## Footnotes

^1^We do acknowledge that the use of different colors in the learning task cannot prevent extinction completely. If learning of reward-associations transfers from the learning task to the search task to influence attentional capture, of course learning about the omission of reward in non-reinforced search trials could transfer back to the learning task. However, the use of different colors was crucial to prevent the transfer of motor preparation processes from the learning task to the search task as further detailed in the “Discussion” Section.

^2^www.xrite.com

^3^www.neurobs.com
