# Supplementary material for: Reward Draws the Eye, Uncertainty Holds the Eye: Associative Learning Modulates Distractor Interference in Visual Search
Source: Front Behav Neurosci. 2017 Jul 11;11:128. doi: 10.3389/fnbeh.2017.00128 (PMC5504121; doi:10.3389/fnbeh.2017.00128)
Supplement: Supplementary file 1 [file Data_Sheet_1.docx]

# Appendix A

Simulations in Figure 1 refer to a classical conditioning experiment in which three conditioned stimuli are followed by reward on 100%, 50% and 0% of the trials.

In economic decision theory expected reward and reward uncertainty are defined as the expected value (Eq. 1) and variance (Eq. 2) of the rewards probability distribution (Schultz et al., 2008). Reward is treated as a random variable λ that can take values λ_0_ = 0 and λ_1_ = 1 with probabilities *p*_0_ and *p*_1_ to represent no reward and reward respectively.

*E*[λ] = λ_0_ *p*_0_ *+* λ_1_ *p*_1_ = λ_1_ *p*_1_ (Eq. 1)

*Var*(λ) = *p*_1_(1 - *p*_1_) (Eq. 2)

The learning rule of Rescorla and Wagner (1972; Wagner & Rescorla, 1972; Eq. 3) computes how the strength of a cue-outcome association *V* changes after each learning episode in proportion to the prediction error of that learning episode. The error is computed as the difference between the actual value of the outcome λ minus the value of the outcome predicted by all cues present Σ*V*. β is a learning rate parameter that depends on the effective strength of the outcome (e. g. amount of food / state of deprivation). α is a learning rate parameter that increases with the physical salience of the cue.

Δ*V* = α · β · (λ – Σ*V*) (Eq. 3)
